# Prevalence and correlates of sleep disturbance among adolescents in the eastern seaboard of China

**DOI:** 10.1186/s12889-024-18564-0

**Published:** 2024-04-10

**Authors:** Haidong Yang, Lingshu Luan, Jiuli Xu, Xingran Xu, Xiaowei Tang, Xiaobin Zhang

**Affiliations:** 1 Department of Psychiatry, The Fourth People’s Hospital of Lianyungang, The Affiliated KangDa College of Nanjing Medical University, 222003 Lianyungang, P.R. China; 2 Xuzhou Medical University, 221004 Xuzhou, P.R. China; 3 Department of Psychiatry, Affiliated WuTaiShan Hospital of Medical College of Yangzhou University, 225003 Yangzhou, P. R. China; 4 Suzhou Psychiatric Hospital, Institute of Mental Health, The Affiliated Guangji Hospital of Soochow University, 215137 Suzhou, P.R. China

**Keywords:** Sleep disturbance, Suicidal ideation, Adolescents, PHQ-9, GAD-7, PSSS

## Abstract

**Background:**

Sleep disturbances are serious public health issues that warrant increased attention, especially in adolescents. The aim of this study was to investigate the prevalence and factors associated with sleep disorders among urban adolescents in China.

**Methods:**

This study utilized an online survey to assess the demographic characteristics and mental health status of secondary school students in Lianyungang City. The Patient Health Questionnaire-9 (PHQ-9) was used to evaluate sleep disturbances in adolescents. The seven-item Generalized Anxiety Disorder (GAD-7) assessed anxiety symptoms, and the Perceived Social Support Scale (PSSS) was used to measure perceived social support.

**Results:**

Among 3443 adolescents, the prevalence of sleep disorders were 10.8%, with significantly higher proportions of sleep disorders (13.7% VS 8.3%, *P* < 0.001) among female adolescents when compared to males. Binary regression analysis revealed that anxiety symptoms (OR = 1.305, 95% CI: 1.269–1.342, *P* < 0.001) was risk factor for sleep disturbances, and significant other support (OR = 0.944, 95% CI: 0.896–0.994, *P* = 0.028) and good annual household income (OR = 0.616, 95% CI: 0.394–0.963, *P* = 0.034) were protective factors. Furthermore, multinomial logistic regression analysis showed that age, sex, and anxiety symptoms were associated with an elevated risk of experiencing more frequent sleep disturbances (all *P* < 0.05).

**Conclusions:**

We have found that 10.8% of adolescents experience sleep disorders, and it is evident that various factors can influence healthy sleeping. These results underscore the significance of addressing these factors to enhance sleep health among this population.

## Introduction

Among the array of issues which adolescents may face due to physiological, psychological, and social factors, sleep is increasingly emerging as a salient concern. Research has indicated that high-quality sleep plays a paramount role in maintaining optimal cognitive and emotional states among adolescents [1–3]. Sleep insufficiency can lead to a variety of issues, including difficulty concentrating, reduced academic performance, obesity, heightened suicidal thoughts, and in some cases, an elevated risk of car accidents, workplace injuries, and sports-related injuries [4, 5]. Regrettably, sleep disturbances have been increasingly prevalent among adolescents worldwide [6]. A recent systematic review and meta-analysis revealed that sleep disturbances greatly impacted children and adolescents during the COVID-19 pandemic, making them the second most vulnerable population, with an overall combined prevalence of 45.96% [7]. A systematic review and meta-analysis reported that sleep disorders among Chinese adolescents had an overall pooled prevalence of 26% [8], in the general population the prevalence of sleep disturbance was 15% [9]. A cohort study conducted in Germany, focusing on adolescents aged 10–17 years, found that approximately 20% of the participants exhibited sleep-related problems [10].

Sleep disturbances, in addition to symptoms of depression and anxiety, are a significant concern affecting adolescents [11, 12]. There is an association between sleep disturbances and the onset of depression or psychiatric disorders in adolescents or early adulthood, with this association being specific to the type of sleep disturbance and clinical diagnostic criteria [13]. Ceouse et al. reported that the instability of circadian rhythms during adolescence, such as delayed sleep-wake phases, combined with environmental factors—including but not limited to changes in daily routines and exposure to nighttime light—may exacerbate the misalignment between the biological clock and external timing, which in turn could promote or lead to the onset of depression during adolescence and early adulthood [14]. A long-term longitudinal follow-up study found that the use of composite measures of sleep disturbances (including insomnia and hypersomnia symptoms, insomnia and impaired daytime functioning, or hypersomnia and anergia) was associated with the risk of depression and hypomania in adolescents and young adults [15]. Although there is an association between subjective sleep disturbances and depressive symptoms in adolescents, it is important to note that the strength of the relationship varies across different types of sleep disturbances and depressive symptoms [16]. Overall, sleep disturbances have a negative impact on adolescents and should be recognized early and actively prevented.

Furthermore, several factors may be associated with sleep disturbances among adolescents, including family or academic pressures and problematic internet use [17, 18]. Previous research has demonstrated that family economic hardship has a detrimental impact on sleep quality in adolescents [19]. Academic and social pressures from peers have also been found to be strongly linked to sleep disturbances and an elevated risk of experiencing suicidal thoughts [20, 21]. The results of the influence of age on sleep disturbances were inconsistent. For example, Liang et al. found that the prevalence of sleep disorders was low in junior high school and increased in senior high school [8], whereas Cai et al. found that younger children were associated with a higher risk of sleep disturbances [22]. A meta-analysis of longitudinal studies showed that while sleep disturbance had a weak association with suicidal thoughts and suicidal behavior, insomnia emerged as a significant predictor of suicidal ideation, and nightmares were strongly linked to suicide attempts [23]. Individually and in combination, these factors have a profound impact on the mental health of adolescents. It is crucial to comprehend their intricate interactions to effectively address and minimize the risks associated with sleep disorders.

Understanding the prevalence and factors related to sleep disturbances among adolescents was vital for the development of effective preventive interventions. Therefore, the primary objective of the present study was to investigate (1) the prevalence of sleep disturbances among adolescents and (2) identify potential risk factors associated with varying severity of sleep disturbances. We hypothesized that there is an increased prevalence of sleep disturbances among adolescents. Furthermore, we postulated that differing severity of sleep disturbances may be linked to various sociodemographic and behavioral factors.

To the best of our knowledge, there is currently little research regarding the prevalence and risk factors associated with different severity of sleep disturbances among adolescents in China.

## Methods

### Procedures and subjects

We conducted a cross-sectional survey between May and June 2023 to collect data from secondary school students in the city of Lianyungang, Jiangsu Province, China. The sampling process involved assigning identification numbers to the secondary schools in the urban area of Lianyungang and using a lottery method to randomly select 5 schools. Unbiased school teachers, who were not affiliated with the research team, then carried out a random selection of students from these 5 schools to guarantee the independence, blindness, and randomness of participant selection. The participants were drawn from various grades, spanning from 7th grade to 3rd year of high school. The survey was administered online using the Wenjuanxing platform (https://www.wjx.cn/app/survey.aspx) and the study received approval from the Ethics Committee of Lianyungang Fourth People’s Hospital. The survey was conducted with the consent of the school, parents, and students. Prior to the survey, the students were given comprehensive information about the purpose, process, data confidentiality, and anonymity of the survey. Their participation was entirely voluntary, and they had the option to discontinue completion of the questionnaire at any time.

### Socio-demographic information

The research team designed a semi-structured questionnaire to gather sociodemographic data, which included information on age, gender, single-parent households (yes/no), parental relationships (harmonious/moderate/troubled), and annual household income (better/good/fair/poor). Considering the comprehension of the parental relationship by secondary school students, the parental relationship was defined using a question: “What do you think is the relationship between your father and mother?” There were three responses: harmonious (parents have a better relationship with each other, with fewer quarrels), moderate (parents have a good relationship with each other, with occasional quarrels) and troubled (parents have a poor relationship with each other, with frequent quarrels).

Based on the Statistical Bulletin of National Economic and Social Development of Lianyungang City in 2022, we designed a multiple-choice question in the questionnaire, which contains 4 options, the annual household income is: A less than ¥50,000, B ¥50,000-100,000, C ¥100,000-200,000, D more than ¥200,000. Options A, B, C, and D correspond to poor, fair, good, and better, respectively.

### Measurements

#### Sleep disturbances

The 9-item Patient Health Questionnaire (PHQ-9) is a commonly used self-assessment questionnaire in which participants respond to nine questions to assess their individual mental health status over the past two weeks, often in primary care clinical settings [24]. The PHQ-9 consists of nine items, with each item rated on a scale of 0 to 3. The scoring scale is as follows: 0 for “not at all”, 1 for “several days”, 2 for “more than half the days”, and 3 for “nearly every day”. We assessed the presence of a sleep disturbance by utilizing the third item “Trouble falling or staying asleep, or sleep too much” of the PHQ-9 questionnaire, respectively. A cut-off score of 0–1 signifies no sleep disorders, while a cutoff score of 2–3 indicates their presence [25].

#### Anxiety symptoms

The 7-item Generalized Anxiety Disorder Scale (GAD-7) is a widely used self-assessment scale consisting of seven items. It helps evaluate an individual’s anxiety symptoms experienced within the last two weeks [26, 27]. A 4-point scale ranging from 0 to 3 was utilized to score each item on the GAD-7. The total score ranges from 0 to 27, where higher scores indicate more severe anxiety symptoms. On the GAD-7 scale, the ratings 0, 1, 2, and 3 correspond to the following descriptions: “not at all”, “several days”, “more than half the days”, and “almost every day”, respectively [26]. These ratings are used to assess the frequency of anxiety symptoms experienced by an individual. Spitzer et al. found that the sensitivity and specificity of the GAD-7 scale were 89.0% and 82.0% [26]. A study has shown that the GAD-7 has good reliability and validity in evaluating anxiety symptoms among Chinese adolescents [28].

#### Perceived social support

Perceived social support was assessed using the Chinese version of the Perceived Social Support Scale (PSSS), which comprises 12 items categorized into three subscales: family (items 3, 4, 8, and 11), friends (items 6, 7, 9, and 12), and significant other (items 1, 2, 5, and 10) [29, 30]. Each item was rated on a 7-point scale, ranging from “very strongly disagree” to “very strongly agree,” with higher scores indicating a greater level of perceived social support for the individual [31]. The Cronbach’s alpha coefficients for the subscales of family, friends, and significant other, as measured by Zimet et al., were 0.87, 0.85, and 0.91, respectively [31]. The scale has demonstrated robust psychometric properties and has been validated in Chinese populations, indicating its reliability and validity [32].

### Statistical analyses

Normality distribution tests were performed using Kolmogorov-Smirnov for continuous variables, such as age, comparisons were performed using either the Student’s *t*-test or independent samples t-test, with mean and standard deviation (SD) as measures of central tendency and dispersion, respectively. Categorical variables, such as percentages or frequencies, were compared using descriptive statistics and the chi-square test. Post hoc tests were performed using Bonferroni-correction, which was done by multiplying the *p*-value from each comparison by the total number of comparisons conducted. Univariate analyses were performed to identify the risk factors associated with sleep disturbance, incorporating binary logistic regression. We categorized the presence of sleep disorders as a dichotomous variable and employed binary logistic regression analyses to assess the relationship between sleep disorders and other variables to analyze associated risk factors. We conducted multinomial logistic regression analyses to assess the risk factors for sleep disturbances. Sleep disturbances were categorized into mild (1 point), moderate (2 points), and severe (3 points) based on the scores. We used these categorizations to evaluate the risk factors that might be linked to the severity of sleep disturbances. To ensure the study has adequate statistical power, we utilized G*Power 3.1 software for sample size estimation and determined the minimum required sample size. The calculation considered the anticipated effect size (0.80), α level (0.05), and power (1-β, 0.8). For the Ratio var1/var0, we selected a conservative estimate of 0.9, resulting in a minimum required sample size of 1436. All statistical analyses were performed using the Statistical Package for the Social Sciences (SPSS) software, version 23.0. A two-sided P-value of less than 0.05 was considered statistically significant.

## Results

### Sociodemographic characteristics of participants

A total of 3532 questionnaires were collected, with 89 (2.52%) excluded due to incomplete information on age, sex, grade, and other required fields. Ultimately, a total of 3443 valid questionnaires were obtained. The age range of the adolescents who participated in the survey was from 12 to 18 years old, with an average age of 14.88 (1.59) years. The average total score on PSSS was 65.0 (15.18) and the overall prevalence rates among the surveyed population were 10.8% (*n* = 373) for sleep disturbances. Overall, the percentages for parental relationships were 72.0% (*n* = 2479), 24.2% (*n* = 832), and 3.8% (*n* = 132) for harmonious, moderate, and troubled, respectively. Single-parent households accounted for 10.0% (*n* = 346) of the total. For annual household incomes, the percentages were 25.4% (*n* = 876), 20.1% (*n* = 20.1), 51.9% (*n* = 1786), and 2.6% (*n* = 89) for better, good, fair, and poor, respectively.

### Comparison of adolescent characteristics with or without sleep disturbances

The results of the self-report indicated that there were a higher proportion of female adolescents with sleep disorders than males (χ^2^ = 26.599, *P* < 0.001). Additionally, a significant difference was found between individuals with and without anxiety symptoms in individuals of sleep disturbances (χ^2^ = 893.818, *P* < 0.001). Adolescents without sleep disorders reported significantly higher PSSS scores in terms of family, friends, and significant other when compared to those with sleep disorders (all *P* < 0.001). Adolescents from single-parent families showed a higher prevalence of sleep disorders when compared to those from non-single-parent families (χ^2^ = 14.0, *P* < 0.001). Moreover, adolescents from families with harmonious parental relationships exhibited lower rates of sleep disorders in contrast to those from families with troubled parental relationships (χ^2^ = 133.748, *P* < 0.001). The prevalence of sleep disorders among adolescents from families with higher annual incomes was significantly lower when compared to adolescents from families with lower annual incomes (χ^2^ = 35.889, *P* < 0.001), as shown in Table 1. Subsequently, categorization based on the mean scores of family, friends, and significant other on the PSSS as a threshold revealed that the number of adolescents with sleep disorders who received support from family, friends, and significant other were 195 (22.9%), 214 (19.6%), and 192 (23.6%), respectively.

Furthermore, of the 3,443 adolescents, the percentages of self-reported sleep disorders with scores of 0, 1, 2, and 3 were 63.6%, 25.5%, 6.2%, and 4.7%, respectively.

**Table 1:** Socio-demographic profile of participants with and without sleep disturbances (*n* = 3443)

Variable	sleep disturbances			
	With (*n* = 373)	Without (*n* = 3070)	χ2/t	*P*
Age (years), mean (SD)	14.63 (1.50)	14.91 (1.60)	3.234	0.001
Sex, n (%)			26.599	< 0.001
Male	150 (8.3)	1668 (91.7)		
Female	223 (13.7)	1402 (86.3)		
GAD, mean (SD)	10.84 (5.99)	2.89 (3.67)	25.034	< 0.001
PSSS, mean (SD)				
Family	17.10 (5.97)	22.32 (5.30)	16.165	< 0.001
Friends	18.46 (5.82)	21.98 (5.11)	11.155	< 0.001
Significant other	17.48 (5.70)	22.15 (5.06)	15.096	< 0.001
Single-parent households, n (%)			14.000	< 0.001
Yes	58 (16.8)	288 (83.2)		
No	315 (10.2)	2782 (89.8)		
Parental relationship, n (%)			133.748	< 0.001
Harmonious	189 (7.6)	2290 (92.4)		
Moderate	138 (16.6)	694 (83.4)		
Troubled	46 (34.8)	86 (65.2)		
Annual household income, n (%)			35.889	< 0.001
Better	79 (9.0)	797 (91.0)		
Good	49 (7.1)	643 (92.9)		
Fair	223 (12.5)	1563 (87.5)		
Poor	22 (24.7)	67 (75.3)		

SD, standard deviation; GAD, Generalized Anxiety Disorder-7; PSSS, Perceived Social Support Scale.

### Correlation analysis of the prevalence of sleep disturbances in adolescents

Significant relationships were observed between age, sex, anxiety symptoms, PSSS family, friends, and significant other, single-parent families, parental relationships, and annual household income, and sleep disturbances according to univariate analyses (all *P* < 0.05). Consequently, to account for these findings, the aforementioned variables were included in the binary logistic regressions. In addition, univariate analysis showed a significant association between total PSSS score and sleep disturbance (B = -0.052, *P* < 0.001, OR = 0.949, 95%CI: 0.943–0.956). The results revealed anxiety symptoms (OR = 1.305, 95% CI: 1.269–1.342, *P* < 0.001) to be risk factors for sleep disturbances in adolescents. Significant other support and good annual household income were protective factors for sleep disturbances (OR = 0.944, 95% CI: 0.896–0.994, *P* = 0.028; OR = 0.616, 95% CI: 0.394–0.963, *P* = 0.034), respectively. However, age, sex, family, and friend support, single-parent households, and parental relationship were not risk factors for sleep disturbances (all *P* > 0.05), as shown in Table 2.

Further, the correlation matrix revealed that family showed a positive correlation with friends (*r* = 0.070) and a negative correlation with significant other (*r* = − 0.599), while friends showed a negative correlation with significant other (*r* = − 0.647).

**Table 2:** Risk factors associated with the prevalence of sleep disturbances

Variables	B	OR	95%CI	*P*
Age	-0.041	0.960	0.881–1.046	0.353
Sex	0.253	1.288	0.986–1.682	0.063
GAD	0.266	1.305	1.269–1.342	< 0.001
PSSS				
Family	− 0.026	0.975	0.937–1.014	0.205
Friends	0.036	1.037	0.996–1.079	0.074
Significant other	− 0.058	0.944	0.896–0.994	0.028
Single-parent households	0.144	1.155	0.748–1.782	0.516
Parental relationship				
Harmonious		1		
Moderate	0.126	1.134	0.831–1.547	0.427
Troubled	0.469	1.598	0.882–2.896	0.122
Annual household income				
Better		1		
Good	− 0.484	0.616	0.394–0.963	0.034
Fair	− 0.102	0.903	0.646–1.263	0.553
Poor	0.000	1.000	0.479–2.084	0.999

GAD, Generalized Anxiety Disorder-7; PSSS, Perceived Social Support Scale.

### Factors related to sleep disturbance severity

Having failed the parallel test, a multinomial logistic regression analysis was conducted to examine the risk factors associated with varying severities of sleep disorders. We used a sleep disturbance score of 1 as a control. As shown in Table 3, compared to adolescents with rare sleep disturbances (score of 1), females who experienced sleep disturbances almost every day (score of 3) had a 1.683-fold increased risk of sleep disorders when compared to males (OR = 1.683, 95% CI: 1.185–2.392, *P* = 0.004), for each 1-point increase in anxiety symptom score, the risk of sleep disorders increased 1.068-fold (OR = 1.068, 95% CI: 1.032–1.106, *P* < 0.001), and for each 1-year increase in age, the risk of sleep disturbances decreased by 0.892 fold (OR = 0.892, 95%CI: 0.798–0.998, *P* = 0.045). In comparison, adolescents experiencing sleep disturbances more than half of the days over a 2-week period (score of 2) had a 1.381-fold increased risk of sleep disorders in females than in males (OR = 1.381, 95%CI: 1.016–1.878, *P* = 0.039), for each 1-point increase in anxiety symptom score, the risk of sleep disturbances increased 1.071-fold (OR = 1.071, 95% CI: 1.038–1.106, *P* < 0.001), and for each 1-year increase in age, the risk of sleep disturbances decreased by 0.903 fold (OR = 0.903, 95%CI: 0.819–0.996, *P* = 0.041). Family (OR = 0.935, 95%CI: 0.893–0.980, *P* = 0.005) and significant other (OR = 1.080, 95%CI: 1.011–1.155, *P* = 0.023) were influence factors for sleep disturbance almost every day (score of 3), but not half of the days (score of 2, all *P* > 0.05), friends were neither (all *P* > 0.05). However, our findings indicate that single-parent household were not significant factors for the determination of sleep disturbance severity (*P* > 0.05).

**Table 3:** Risk factor associated with sleep disturbance severity

Dependent variable	Variables	B	*P*	Exp(B)	95%CI
3 points	Age	− 0.114	0.045	0.892	0.798–0.998
	Sex	0.521	0.004	1.683	1.185–2.392
	GAD	0.066	< 0.001	1.068	1.032–1.106
	Family	− 0.067	0.005	0.935	0.893–0.980
	Friends	− 0.041	0.117	0.960	0.912–1.010
	Significant other	0.077	0.023	1.080	1.011–1.155
	Single-parent households	0.120	0.625	1.127	0.697–1.823
2 points	Age	− 0.102	0.041	0.903	0.819–0.996
	Sex	0.323	0.039	1.381	1.016–1.878
	GAD	0.069	< 0.001	1.071	1.038–1.106
	Family	− 0.008	0.719	0.992	0.949–1.037
	Friends	− 0.037	0.117	0.964	0.921–1.009
	Significant other	0.052	0.098	1.053	0.991–1.119
	Single-parent households	− 0.355	0.165	0.701	0.424–1.158

GAD, Generalized Anxiety Disorder-7.

## Discussion

We found that the prevalence of sleep disturbances in Chinese adolescents was 10.8%, and anxiety symptoms, lower significant other support, and poor annual household income were risk factors for sleep disturbances. Furthermore, younger age, female sex, and anxiety symptoms were found to be associated with an elevated risk of experiencing more frequent sleep disturbances.

Our findings also revealed that the prevalence of sleep disorders among adolescents was 10.8%, which was inconsistent with the results of previous studies. For example, the results were lower than the 26% of Chinese adolescents and 15% of the general population previously surveyed [8, 9], and another cross-sectional survey revealed that the prevalence of sleep disorders among adolescents aged 12–18 years was 26.9% [33]. A large sample conducted in the Netherlands found that less than 20% of adolescents reported experiencing sleep disturbances within a month [34], while in Brazil 53% of adolescents were reported to have poor sleep quality [35]. The observed discrepancies in prevalence may be attributable to our study population being drawn from a single region with similar cultural backgrounds and educational systems. Future research should be conducted across a wider geographical area to enhance the generalizability of the findings. Nevertheless, these consistent findings highlight the significance of addressing the prevalence of sleep disorders in adolescents and emphasizes the need for further investigation in the future.

Our results align with previous research highlighting the impact of psychological factors on sleep disturbances [36, 37]. Adolescents who had poor sleep quality were 3.558 times more likely to experience anxiety symptoms when compared to individuals with good sleep quality [38]. Moreover, higher levels of negative coping styles in adolescents with sleep disorders were found to increase the prevalence of anxiety symptoms, whereas higher levels of positive coping styles were associated with a lower prevalence of anxiety symptoms [38]. These findings are consistent with previous research highlighting the bidirectional relationship between sleep disturbances and psychological factors. Moreover, a prior study utilizing a two-sample Mendelian randomized design demonstrated a robust association between sleep disorders and anxiety symptoms [39]. Bian et al. also reported similar findings, further supporting the association between sleep disorders and anxiety symptoms [40]. A global survey indicates that 7.8% of adolescents suffer from anxiety-induced sleep disturbances, with a strong correlation between extended sedentary behavior and increased risk of this condition [6]. A network analysis showed an association between sleep disturbances and anxiety symptoms in secondary school students, and that sleep disturbances may be associated with enormous learning burden [41]. The relationship between anxiety symptoms and sleep disorders is intricate and multifaceted, and further research is needed to understand the underlying mechanisms.

Lower significant other support was risk factor for sleep disturbances among adolescents. In line with previous research, it was clear that perceived social support plays an important role on the development of depressive symptoms, anxiety symptoms, and sleep disorders [42, 43]. A study found that support from friends negatively correlated with sleep duration, while support from family seemed to positively contribute to sleep quality, and the presence of other significant forms of support mitigated the negative impact on sleep duration [44]. Another study indicated that social support has a mediating role between negative emotions and sleep quality, that is, the association between negative emotions and sleep quality decreases with increased social support [45]. Our findings showed that adolescents who perceive more support from family also tend to perceive more support from friends, although this correlation is very weak; they also perceive less support from significant others. Meanwhile, when adolescents perceive more support from friends, they may perceive less support from significant others, and vice versa. We speculated that adolescents may have a trade-off or substitution effect between family, friends, and significant other. Previous study suggested that there might be a supportive and reinforcing relationship between the three dimensions of social support: family, friends, and teachers [46], while positive social relationships are not correlated with sleep quality, and negative social relationships are associated with sleep quality in a bidirectional manner [47], which necessitates further exploration in future studies to deepen the understanding of the interplay between dimensions of social support on adolescent.

Similarly, family economic crises, such as unemployment and financial constraints, have a significant impact on the mental health of adolescents [48]. However, a study conducted in South Korea revealed that adolescents residing in urban areas, as well as those from high-income families, experienced shorter sleep durations and later bedtimes [49]. These findings suggest the importance of social support networks and socioeconomic factors affecting sleep.

In line with previous studies, our results indicate that females are at a higher risk of experiencing more frequent sleep disturbances [50]. This gender difference may be attributed to various biological, psychological, and sociocultural factors. Previous reviews have indicated that pubertal development, hormonal changes, genetic mechanisms, gestational period, and mode of birth, as well as behavioral and social environments, contribute to this increased susceptibility [51, 52]. Regarding the influence of age on sleep disturbances our study was consistent with Cai et al. [22] in that the risk of sleep disturbances progressively decreases with increasing age, while inconsistent with Liang et al. [8]. It is hypothesized that, with advancing age, adolescents experience more stable circadian rhythms and hormonal levels, potentially learn more effective coping strategies and stress management skills, develop better sleep habits, and gain increased autonomy over sleep regulation, which, collectively through a series of biological, psychological, and behavioral changes, may contribute to a reduced incidence of sleep disorders. However, the exact mechanisms underlying this association remain unclear.

Interestingly, our findings suggested that parental relationship was not an influencing factor for adolescent sleep, which was inconsistent with previous findings. A systematic review study revealed that the dissolution of parental relationships (such as divorce or separation) has negative effects on the sleep of children and adolescents aged 0 to 18 years [53].The other longitudinal study suggested a bidirectional relationship between adolescents’ sleep quality and family factors, with changes in sleep quality potentially impacting family dynamics, although the association between sleep quality and family relationships (support or conflict) was small but non-significant [54]. Dysfunctional relationships between parents also contribute to increased insecurity among adolescents, leading to anxiety and a higher likelihood of experiencing sleep disturbances. Our findings indicated that only 132 of the participating adolescents reported poor parental relationships, a relatively low number that may be related to adolescents’ capacity to observe parental relationships, comprehension, insight, and comprehension of the definition of “parental relationship”, as well as imbalances in the distribution of choices that may affect the reliability of the results, and that need to be further investigated.

Recognizing the importance of addressing sleep disturbances among adolescents, healthcare professionals and policymakers should emphasize early identification and intervention strategies. By understanding the underlying mechanisms that link risk factors and sleep disturbances, targeted interventions can be developed to improve the mental well-being and overall health of this vulnerable population. Future research should focus on examining these mechanisms and developing effective strategies to enhance sleep quality.

Despite the significant findings and contributions derived from our study, there were several limitations that should be acknowledged. First, cross-sectional design limits establishing causality between risk factors and sleep disturbances; longitudinal studies are needed to unravel temporal relationships. Second, despite previous studies using the PHQ-9, GAD-7, and PSSS scales to evaluate adolescents’ mental health, the appropriateness of these instruments still requires further investigation. Moreover, the tools for collecting demographic information regarding parental relationships and economic status also need additional refinement. Third, the study sample focused exclusively on adolescents in one area, limiting generalizability; therefore, research in diverse populations is needed to determine the complex interplay between cultural and individual factors. Fourth, the use of convenience sampling rather than stratified sampling may lack representativeness, which could bias the results, and future studies will need more comprehensive sampling methods to enhance the representativeness of the findings. Finally, ethical and methodological issues arising from the survey, such as participants’ understanding of parental relationships and household economic situations, as well as the emotional impact of these sensitive issues on their responses, may affect the authenticity of the results. Therefore, future research should incorporate more qualitative research methods, enhance interaction between participants and researchers, and further explore and improve ethical protective measures to ensure the accuracy and reliability of survey results.

In summary, our study provided valuable insights into the prevalence and risk factors associated with sleep disturbances among adolescents, where anxiety symptoms, lower significant other support, and annual household income were identified as risk factors. Furthermore, younger age, female sex, and anxiety symptoms were found to be associated with an elevated risk of severe sleep disturbances. These findings highlight the need for comprehensive strategies to address sleep disturbances in adolescents, focusing on early detection, mental health support, and promoting positive social and familial relationships.

## Data Availability

The datasets used and analyzed during the current study are available from the corresponding author on reasonable request.
